# Uniportal Video-Assisted Thoracoscopic Surgery for Minor Procedures

**DOI:** 10.3390/jpm14080880

**Published:** 2024-08-20

**Authors:** Apostolos C. Agrafiotis, Sotirios D. Moraitis, Georgios Sotiropoulos

**Affiliations:** 1 Department of Thoracic and Vascular Surgery, Wallonie Picarde Hospital Center (Centre Hospitalier de Wallonie Picarde—CHwapi), 7500 Tournai, Belgium; 2 Department of Thoracic Surgery, Saint-Pierre University Hospital, 1000 Brussels, Belgium; 3 Department of Thoracic Surgery, Athens Naval and Veterans Hospital, 115 21 Athens, Greece

**Keywords:** uniportal, VATS, pleural empyema, pneumothorax

## Abstract

Introduction: Uniportal video-assisted thoracoscopic surgery (uVATS) is becoming popular for major lung resections, even for more complex procedures. The technique initially described for minor procedures seems more difficult to reproduce and has a longer learning curve. This review aims to describe the evolution from multiportal to uVATS and to explore its feasibility and reproducibility by identifying its drawbacks and limitations. Methods: Research from PubMed was obtained with the terms [uniportal] AND [surgery] OR [single-port] AND [thoracic surgery] OR [VATS]. Papers concerning pediatric cases and non-English papers were excluded. Individual case reports were also excluded. Discussion: uVATS seems to be widely adopted and performed for minor procedures. The applicability of uVATS for different indications is discussed, even though practically all thoracic surgical interventions can be performed through a single incision. Conclusions: The transition from conventional three-port VATS to uVATS is described in this paper. An increasing number of thoracic surgeons worldwide have adopted this approach, even for major complex anatomical lung resections. Regarding the performance of minor thoracic interventions, we believe this technique is easily reproducible with a short learning curve because the instruments do not cross each other, and intraoperative movements remain intuitive. It is therefore a feasible, safe, and efficacious technique. For these reasons, we believe uVATS should be offered to all patients undergoing minor thoracoscopic procedures.

## 1. Introduction

Compared to typical open thoracotomy, video-assisted thoracoscopic surgery (VATS) has been shown since its introduction in the early 1990s to reduce pain dramatically, speed recovery, minimize complications, and improve postoperative quality of life for patients requiring thoracic surgery. Nowadays, nearly all general thoracic operations are performed via the standard three-port VATS technique [1].

Indeed, the first procedures that a trainee in general thoracic surgery deals with are minor diagnostic and curative procedures (cure of spontaneous pneumothorax, pleural biopsy, pulmonary biopsy, pericardial window, chemical pleurodesis, wedge resection of superficial nodules, etc.) performed traditionally using three-port VATS. The surgeon can advance to anatomical VATS resections when sufficient experience is acquired with minor VATS procedures.

Thoracic surgeons have continued to enhance patient outcomes through innovation, improvisation, and the integration of novel procedures and technologies. This has led to the development of several more advanced VATS approaches, which could further minimize the size and number of incisions [1,2,3].

While surgeons have become more experienced and proficient with three-port VATS, the number of operative ports has decreased. Consequently, during the last decade, there has been a recrudescence of uniportal video-assisted thoracoscopic surgery (uVATS). Similarly, with conventional VATS’s evolution, nearly all thoracic surgical procedures are now performed by uVATS. This review aims to describe the evolution from multiportal to uVATS and explore its feasibility and reproducibility by identifying its drawbacks and limitations.

## 2. Methods

The current recommendations for the quality assessment of narrative review articles were followed in conducting this review [4]. Research from PubMed was obtained using the terms [uniportal] AND [surgery] OR [single-port] AND [thoracic surgery] OR [VATS]. Papers concerning pediatric cases and non-English papers were excluded. Individual case reports were also excluded. Papers were chosen based on relevance because the current study is not a systematic review. Most studies were retrospective case series, and, consequently, papers with higher levels of evidence were not identified. The references of selected papers were sought to find other pertinent articles.

## 3. The Evolution from Multiportal to Uniportal VATS

The framework used in conventional VATS is consistent with the configuration of open surgery. The camera takes the place of a direct view through the wound, and instruments replace the hands of the surgeon. This eliminates the need for rib spreading and permits the use of three small ports to perform the same thoracic surgical procedure [1]. It is now generally accepted that the majority of the discomfort and morbidity related to open thoracotomy is caused by the spreading of the ribs [5]. Although nonrandomized trials have indicated that VATS lowers postoperative morbidity, there is no solid proof that it is better than open surgery. Although early studies indicated that VATS was associated with less postoperative pain, better quality of life, shorter hospital stay, and faster recovery and resuming of daily activities, some studies did not show any superiority over open thoracotomy [6,7,8] More specifically, a randomized prospective trial published in 1995 failed to show any advantage of VATS lobectomy [7]. The outcomes of thirty patients who underwent muscle-sparing thoracotomy were compared to those of twenty-five patients operated using VATS. The VATS approach did not significantly decrease the duration of chest tube drainage, length of hospital stay, post-thoracotomy pain, or recovery time and resumption of professional activities [7]. For that reason, skepticism was inevitable among the surgical community about the clinical value of VATS surgery. In what followed, VATS passed through many stages and landmarks to establish itself not as an alternative but as the preferred surgical access for early-stage lung cancer [9]. The initial experience mainly derived from case reports led to the publication of more extensive case series. Then, as more robust data were provided and more well-designed large-scale studies were conducted, VATS became the standard of care for early-stage lung cancer [10]. The decrease in the cost of surgical instruments from production to better management in the operating room certainly rendered the application of new techniques more affordable for institutions and surgical teams [11,12].

The initially described multiportal VATS technique used a “diamond” configuration to reproduce triangulation [1]. The camera was inserted through the inferior port, typically in the seventh or eighth intercostal space in the mid-axillary line. The other two ports that permitted the insertion of instruments were placed superiorly to the first one in a triangular pattern, in the fourth or fifth intercostal space anteriorly, and just in front of the tip of the scapula posteriorly [1,2]. The next step was the adoption of a modified configuration, with the camera port being displaced anteriorly in the anterior axillary line. The posterior port was placed in a more inferior position to improve ergonomics. The position of the anterior port was not modified. As experience was gained with that technique, the next step was deleting the posterior port. The camera port was not modified, and all the grasping instruments, energy devices, and staplers were introduced through the utility anterior port. As they moved from traditional three-port VATS to two-port VATS, the surgeons became accustomed to introducing all the instruments through the same utility port. From there, the camera was integrated into the utility port to create a uniportal configuration [1].

Reinersman et al. described the past and present of uVATS [13]. According to the authors, the introduction of this approach has been associated with the development of improved visualization hardware, articulated staplers, and instruments. Indeed, in 2004, Rocco et al. described their initial experience with uVATS [14]. Since then, they published their 10-year experience with 644 patients [15]. Rocco et al. focused on using articulated instruments to reproduce the classical triangulation [14]. The authors required an instrument rotation of 90° along the sagittal plane. They advised crossing the instruments outside the patient to reproduce triangulation inside. In our opinion, these maneuvers are too complicated to be reproducible and could be the reason for the limited use of this technique. In 2013, Gonzalez-Rivas published their two-year experience with uVATS, including 102 patients who underwent lobectomy [16]. Since then, they used this approach to perform more complex resections such as segmentectomy or bronchoplastic resections [17]. The use of uVATS for major lung resections was debatable in the past as it was acknowledged that a new technique should be safely and easily reproducible to be widely developed [18]. However, this debate seems obsolete nowadays because the number of surgeons familiar with these techniques is constantly increasing. In 2014, Song et al. reported their initial experience with uVATS in 264 cases. There were various indications; among them, primary spontaneous pneumothorax was the most frequent. Other indications were secondary spontaneous pneumothorax, lung and pleura biopsy, mediastinal lymph node biopsy, mediastinal mass excision, empyema decortication, lobectomy, pulmonary metastasectomy, pericardial window formation, and hematoma evacuation. Of these, 237 cases underwent uVATS successfully. An additional incision was needed in 10.2% of the cases. The reasons for conversions were extensive pleural adhesions, difficulty with endoscopic stapling, bleeding, and intolerance of one-lung ventilation. The conversion rate of empyema was 54.5%. In contrast, the conversion rate of primary spontaneous pneumothorax was 4.7%, which was the more suitable indication for uVATS according to the authors. In conclusion, the authors acknowledged the feasibility and safety of uVATS for most thoracic procedures; however, they outlined its limitations, especially in the case of complicated pleural empyema [19]. One other significant issue that was brought up concerning the uVATS approach was the possibility of higher surgical expenses as a result of the usage of articulating disposable devices. Salati et al., however, assessed these variables in 51 consecutive patients receiving primary spontaneous pneumothorax treatment using either uVATS or three-port VATS. They demonstrated that the expenses for surgical supplies and operating room expenses were similar for the two groups. On the other hand, patients receiving the uniportal approach experienced a considerable reduction in postoperative costs overall due to their much shorter hospital stays (3.8 vs. 4.9 days) [13,20]. The cost-effectiveness of the uVATS approach was also demonstrated in the retrospective study of Mizukami et al. [21].

From the above, it is evident that uVATS has been widely adopted and performed for minor procedures. However, there is no robust evidence demonstrating its superiority over conventional VATS. A recently published randomized controlled trial showed that uVATS was superior regarding operative time, blood loss, pleural drainage, length of stay, and the average postoperative pain score [22]. However, the sample size was small, as 18 patients were enrolled in each group.

In the following sections, the applicability of uVATS for different indications is discussed, even though, as already mentioned, practically all thoracic surgical interventions can be performed through a single incision.

### 3.1. uVATS for the Treatment of Spontaneous Pneumothorax

Many authors have suggested uVATS as a first-line approach, especially for spontaneous pneumothorax [3,23,24,25]. Technological advancements have provided new surgical instruments to facilitate lung resection through a single incision [26]. In the series published by Masmoudi et al., the cutaneous incision was small but the intercostal space was opened widely in the inside, which avoided the crowding of instruments and enabled better maneuverability [3]. There have been attempts to compare uVATS and multiportal VATS, with many studies focusing on the surgical treatment of spontaneous pneumothorax. A best-evidence topic published in 2015 showed no differences in most postoperative outcomes between multiportal and uVATS [27]. A meta-analysis published in 2016 concluded that uVATS is associated with less postoperative pain and faster recovery, while the complication and recurrence rates were similar to those of three-port VATS, in accordance with another meta-analysis focused on pneumothorax surgery, which was published a year earlier [28,29]. On the other hand, some authors found that the benefit of uVATS was small, as they suggested that early chest tube removal was the most important cause of pain reduction and not using a single port [30].

### 3.2. uVATS for Wedge Resections and Pulmonary Biopsies

Thoracic surgeons can easily resect pulmonary lesions via uVATS [31]. Either superficial or more deeply situated lesions can be resected; the latter may need a preoperative marking. The lesions can be benign or malignant. Mizukami et al. retrospectively compared 145 consecutive patients who underwent wedge resections either with conventional VATS or uVATS. The frequency of epidural anesthesia was significantly higher and the operative time was significantly longer in the multiport group. The number of stapler cartridges, the duration of pleural drainage, and the occurrence of postoperative complications did not differ significantly between groups. According to the authors, the pain control was better in the uVATS group [21].

Pulmonary biopsies can also be performed to diagnose interstitial lung diseases. In this context, performing uVATS without classic orotracheal intubation (non-intubated VATS) yields better postoperative outcomes, especially in patients with impaired lung function [32].

### 3.3. uVATS for Pleural Biopsies and/or Pleurodesis

Performing pleural biopsies for benign or malignant conditions is a straightforward uVATS procedure. In case of malignant disease, talc poudrage can also be realized. There is no need for three incisions and ports because all instruments are on the same viewing plane. The use of a 30° thoracoscope and curved instruments compensates for the absence of triangulation. There are no specific technical considerations; the same steps as for a conventional three-port VATS are followed. A chest tube is introduced in the pleural cavity through the same incision at the end of the procedure. Some authors also proposed performing decortication for trapped lung using uVATS [33].

### 3.4. uVATS for the Treatment of Pleural Empyema

The utility of uVATS can be extended to the treatment of pleural empyema. Conventional VATS has been proven superior to medical treatment for early-stage and more complex cases [34]. A complete debridement/decortication can be carried out through a single incision; however, the need for additional chest tubes has to be taken into consideration. It has to be noted that early and aggressive surgical treatment of empyema can yield better outcomes and is associated with lower conversion rates. In addition, morbidity and mortality are lower, recovery is faster, the length of stay is shorter, and hospital costs are decreased [35]. For these reasons, the minimal invasiveness of VATS made surgery a first-line treatment of pleural empyema. Even with a single incision, loculated effusions can be drained, and the lung can be completely freed in cases of fibrinopurulent empyema. Indeed, in a retrospective study assessing 35 patients with stage II and III pleura empyema who underwent VATS, the authors concluded that uVATS offers more benefits than thoracotomy and conventional VATS [36]. It gives the surgeon access to every part of the pleural cavity and provides ample room for various surgical maneuvers, optimal visibility, and safety. Like thoracotomy, debridement and decortication yield more precise and secure results with this method. Because uVATS allows for a more straightforward and low-risk conversion rate than standard VATS, it must be considered the first choice in stage II and III empyema when the required surgical experience has been attained [37]. Other groups have demonstrated that decortication of tuberculous pleural empyema seems feasible and effective with uVATS, causing less postoperative pain than three-port VATS [38,39].

### 3.5. uVATS for the Creation of a Pericardial Window

Another straightforward procedure that can be performed with uVATS is the creation of a pericardial window in patients with recurrent pericardial effusions. Rocco et al. described the technique in 2006 [40]. A single 2.5 cm incision is made in the fifth intercostal space along the axillary line, either with the patient in the supine position or with the targeted hemithorax elevated (45°) by an axillary roll. After inserting a 2.7 or 5 mm video-thoracoscope, the pleural cavity is examined. Pericardiocentesis is used to remove a small volume of fluid from a target location that is chosen anterior to the phrenic nerve. Using thin forceps, the parietal pericardium is raised cranially, and endoscopic scissors inserted parallel to the thoracoscope are used to open it. Pericardial incisions made circumferentially complete the window. After the procedure, a chest tube is placed through the same incision. More recently, it was reported that the creation of a pericardial window with uVATS without orotracheal intubation is a diagnostic and therapeutic alternative and might be safely employed in all patient groups with tamponade or pericardial effusion. There are demonstrated benefits to this method, particularly for high-risk surgical patients [41].

### 3.6. uVATS for Thoracic Sympathectomy

Thoracic sympathectomy for primary hyperhidrosis and facial blushing is another procedure that can be safely and successfully performed through uVATS [42]. The patients are installed in the semisitting position, with the abduction of the upper limbs permitting a bilateral surgery, without reinstalling or redraping, which represent a time gain. Excellent results were reported [43]. Instrumentation is conducted either through the utility incision, with instruments being introduced individually, or through the use of a special port conceived for single-incision laparoscopic surgery. Other teams perform this surgery with the instrument that is traditionally used for endoscopic vein harvesting [44]. The use of chest draining after thoracic sympathectomy is not necessary, as simple air evacuation at the end of surgery is sufficient most of the time.

### 3.7. uVATS for the Resection of Lesions Located in the Mediastinum

VATS resection for anterior mediastinal lesions, especially thymic epithelial tumors, is widely accepted and performed as a valid alternative for open sternotomy [45]. The traditional multiportal VATS started to gradually be replaced with uVATS. The choice of entrance in the thoracic cavity is a matter of debate; right-sided, left-sided, or bilateral approaches, even subxiphoid access, seem to be valid options, but each one carries advantages and drawbacks. Depending on the location of the lesion to be resected, the incision is placed in the fourth or fifth intercostal space in the anterior axillary line. In their retrospective study, Wu et al. compared conventional and uVATS in a series of patients with mediastinal tumors irrespective of location. They concluded that compared to traditional VATS, uVATS results in a decreased length of stay and less discomfort following surgery, making it seem like a safe and promising approach [45]. A uniportal technique can also be used to remove neurogenic tumors, pericardial cysts, and bronchogenic cysts from the middle or posterior mediastinum.

Although there are no large-scale prospective studies, uVATS is being increasingly performed for the resection of mediastinal lesions [46,47,48]. The same principles are valid as for conventional VATS; in case of large tumors (>5 cm) and if suspicion of malignancy exists, the resection can be quite challenging through minimally invasive access [48].

### 3.8. uVATS for Biopsy of Mediastinal Lesions and Lymph Nodes

The feasibility of uVATS for the biopsy of mediastinal lesions and lymph nodes has also been investigated [49,50]. In a retrospective cohort assessing 24 uVATS procedures for biopsies of mediastinal lesions, 18 targets involved the anterior mediastinum, while 6 procedures involved the subcarinal or paratracheal region. There were 16 approaches on the right side and 8 on the left side. The diagnostic yield was 100%. The histopathologic analysis of the samples revealed lymphomas in 13 patients, lung cancer in 9, and thymic carcinoma and Castleman disease in 1 patient each [50]. In another cohort, uVATS was performed for lymph node biopsies. Node sampling was performed in the aortopulmonary window, the subcarinal, the right paratracheal, and the paraesophageal station. In all cases, adequate material for pathologic analysis was obtained, showing sarcoidosis, metastasis from non-small-cell lung cancer, and non-Hodgkin’s lymphoma [49]. Both studies reported the feasibility, safety, and efficiency of the uVATS approach.

### 3.9. uVATS for Chest Trauma

The roles and applications of VATS in patients with penetrating thoracic trauma who are hemodynamically stable have been established [51,52,53]. Consequently, uVATS rapidly found its place in that setting [54,55]. A retrospective study reported the experience using uVATS in 19 patients with thoracic stab injuries [56]. The inclusion criteria were the use of a uVATS technique to treat hemodynamically stable patients who had penetrating stab wounds to the chest outside of the cardiac box and who were bleeding actively (output of >1500 mL of blood) after the chest tube was inserted. The patient’s hemodynamic instability, any indication of cardiac or great vascular injury, and any stab wounds inside the chest’s cardiac box were the exclusion criteria [56]. During the procedure, blood and blood clots were removed, and the source of bleeding, which was mainly caused by lung lacerations or intercostal vessels, was located and managed. Electrocautery was utilized to deal with intercostal hemorrhage. Endoscopic staplers were used to perform resection of the injured lung tissues. Another retrospective study demonstrated the feasibility and safety of uVATS [57]. The orifice of the previously inserted chest tube or a separate incision could be used as an operative port. If needed, the incision could be converted into anterolateral thoracotomy. In conclusion, uVATS can be used for exploration, treatment of active bleeding (parenchymal or parietal), lavage of the chest cavity, optimal positioning of the chest tube, and ablation of foreign bodies [58].

### 3.10. uVATS for the Treatment of Thoracic Tuberculous Spondylitis

Xiu et al. enrolled seven patients with thoracic tuberculous spondylitis in their case series [59]. Their study set out to investigate the effectiveness and surgical technique of using uVATS in conjunction with posterior internal fixation for the treatment of thoracic tuberculous spondylitis via debridement and bone graft fusion surgery. There were no serious postoperative complications. According to the authors, compared to previously reported techniques, uVATS causes less trauma and produces satisfactory results.

### 3.11. uVATS for Esophageal Surgery

The surgical treatment of benign esophageal lesions, more particularly esophageal diverticula, through uVATS has been reported [60,61]. Even though only three patients have been operated using uVATS, the operative steps have been well-described and the results seem encouraging. The resection of leiomyomas and the repair of broncho-esophageal fistulas through uVATS have also been reported [62,63].

### 3.12. The Transition from uVATS to uRATS

The emergence and adoption of robotic-assisted thoracoscopic surgery (RATS) provided thoracic surgeons with a new tool that could yield even better results. Inevitably, the RATS approach was inspired by the advances in VATS surgery, giving birth to uniportal RATS, which combines the advantages of both techniques. Even though RATS surgery through a single port is not widely performed yet, studies comparing uVATS to uRATS have shown globally similar postoperative outcomes [64,65]. The increased dexterity in surgical maneuvers that robotic systems provide allows the performance of more complex resections and increases the number of lymph nodes that can be dissected. In one study, uRATS resulted in fewer postoperative complications and decreased the length of hospital stay [64].

## 4. Conclusions

The transition from conventional three-port VATS to uVATS was described in this paper. An increasing number of thoracic surgeons worldwide have adopted this approach, even for major complex anatomical lung resections. As far as the performance of minor thoracic interventions is concerned, we believe that this technique is easily reproducible with a short learning curve because the instruments do not cross each other and intraoperative movements remain intuitive. It is therefore a feasible, safe, and efficacious technique. The list of procedures mentioned in this review, albeit not exhaustive, still demonstrates the tendency to adopt uVATS. All procedures described in this review can be performed through a small incision, which has a cosmetic advantage. For these reasons, in our opinion, uVATS should be offered to all patients undergoing operation for minor thoracoscopic procedures. Even though the available body of evidence is based on retrospective studies, small case series, and case reports, uVATS has generally been accepted as a valid treatment option. It has to be noted that even though the feasibility of uVATS was demonstrated throughout this review, a direct comparison between uniportal and multiportal VATS was not always possible. Some limitations should also be considered, especially in cases of complex pleural empyema, as placing more than one chest tube to optimize pleural drainage may be deemed necessary. The technological advances that guided the recrudescence of conventional VATS (thoracoscopes, articulated instruments, staplers, etc.) will be further improved to accompany the implementation of uVATS by adding new tools to the VATS surgeon’s armamentarium (smaller and flexible thoracoscopes, smaller staplers, wireless instruments) permitting decreases in the mutual interference of instruments inside the single incision, which will inevitably become smaller.

## Data Availability

Not applicable.
